# Make the most of your samples: Bayes factor estimators for high-dimensional models of sequence evolution

**DOI:** 10.1186/1471-2105-14-85

**Published:** 2013-03-06

**Authors:** Guy Baele, Philippe Lemey, Stijn Vansteelandt

**Affiliations:** 1 Department of Microbiology and Immunology, Rega Institute, KU Leuven, Kapucijnen-voer 33 blok I bus 7001, B-3000 Leuven, Belgium; 2 Department of Applied Mathematics and Computer Science, Ghent University, Krijgslaan 281 S9, B-9000 Ghent, Belgium

## Abstract

**Background:**

Accurate model comparison requires extensive computation times, especially for parameter-rich models of sequence evolution. In the Bayesian framework, model selection is typically performed through the evaluation of a Bayes factor, the ratio of two marginal likelihoods (one for each model). Recently introduced techniques to estimate (log) marginal likelihoods, such as path sampling and stepping-stone sampling, offer increased accuracy over the traditional harmonic mean estimator at an increased computational cost. Most often, each model’s marginal likelihood will be estimated individually, which leads the resulting Bayes factor to suffer from errors associated with each of these independent estimation processes.

**Results:**

We here assess the original ‘model-switch’ path sampling approach for direct Bayes factor estimation in phylogenetics, as well as an extension that uses more samples, to construct a direct path between two competing models, thereby eliminating the need to calculate each model’s marginal likelihood independently. Further, we provide a competing Bayes factor estimator using an adaptation of the recently introduced stepping-stone sampling algorithm and set out to determine appropriate settings for accurately calculating such Bayes factors, with context-dependent evolutionary models as an example. While we show that modest efforts are required to roughly identify the increase in model fit, only drastically increased computation times ensure the accuracy needed to detect more subtle details of the evolutionary process.

**Conclusions:**

We show that our adaptation of stepping-stone sampling for direct Bayes factor calculation outperforms the original path sampling approach as well as an extension that exploits more samples. Our proposed approach for Bayes factor estimation also has preferable statistical properties over the use of individual marginal likelihood estimates for both models under comparison. Assuming a sigmoid function to determine the path between two competing models, we provide evidence that a single well-chosen sigmoid shape value requires less computational efforts in order to approximate the true value of the (log) Bayes factor compared to the original approach. We show that the (log) Bayes factors calculated using path sampling and stepping-stone sampling differ drastically from those estimated using either of the harmonic mean estimators, supporting earlier claims that the latter systematically overestimate the performance of high-dimensional models, which we show can lead to erroneous conclusions. Based on our results, we argue that highly accurate estimation of differences in model fit for high-dimensional models requires much more computational effort than suggested in recent studies on marginal likelihood estimation.

## Background

An increasing number of studies in phylogenetics have demonstrated, using a range of different inferential methods of varying complexity, that the assumption of site-independent evolution is overly restrictive and that evolutionary models that take into account context-dependencies may greatly improve model fit. Specifically, context-dependent models are useful when studying mammalian genomes due to the extensive methylation of C in CG doublets, which could make such Cs hotspots for mutation (see [1], for a review). Indeed, more accurate mathematical models of molecular sequence evolution continue to be developed for good reasons as the additional complexity of such models can lead to the identification of important evolutionary processes that would be missed with simpler models. These models however come at a drastically elevated computational cost due to their increase in number of parameters and the need for data augmentation to make the likelihood calculations feasible [2].

The power of Markov chain Monte Carlo (MCMC) techniques enables drawing inference under such complex high-dimensional models in molecular phylogenetics [3]. In this process, comparing alternative models according to objective criteria in a formal model selection procedure is essential in order to select models that best balance simplicity with flexibility and biological realism [4,5]. However, the computational demands associated with increasing model complexity and the amount of data available has considerably hampered careful model assessments. While MCMC approaches cleverly avoid calculating the normalization constant (or marginal likelihood), it is in fact this constant that is of primary importance in model selection (one will choose the model with the highest marginal likelihood). In particular, it is used to calculate the (log) Bayes factor between two models, which is a ratio of two marginal likelihoods (i.e. two normalizing constants of the form *p*(*Y*∣*M*), with *Y* the observed data and *M* an evolutionary model under evaluation) obtained for the two models, *M*_0_ and *M*_1_, under comparison [6]:

(1) $$B_{10}=\frac{p(Y|M_{1})}{p(Y|M_{0})}.$$

Kass and Raftery [7] introduce different gradations to assess the log Bayes factor as evidence against *M*_0_. A value between 0 and 1 is not worth more than a bare mention, whereas a value between 1 and 3 is considered as positive evidence against *M*_0_. Values larger than 3 and 5 are considered to respectively give strong and very strong evidence against *M*_0_. The Bayes factor offers advantages over likelihood-ratio-tests comparing nested models in which one garners evidence only in favor of rejecting less complex models. Instead, the Bayes factor evaluates the relative merits of both competing models and does not require nested models. Further, when the individual (log) marginal likelihoods are estimated correctly, the (log) Bayes factor takes into account differences in dimensions, so higher dimensional models are not automatically preferred.

Several useful methods have been proposed to evaluate marginal likelihoods (and by extension Bayes factors) in phylogenetics, but they are often limited to specific model selection situations, see [8] for an overview. Comparing a few of the methods of potentially general applicability, Lartillot and Philippe [8] test a (simple) Monte Carlo estimator of integrating the likelihood against the model prior and two variants of importance sampling (IS): the posterior harmonic mean estimator (HME) and the stabilized HME to a path sampling (PS) approach [9-11]. Of these approaches, the HME [12] is by far the most popular method in the field of phylogenetics, only requiring samples from the posterior distribution (see e.g. [13]). The HME is however often severely biased and overestimates the true marginal likelihood [14]. Because the HME estimator’s variance may be infinite, a modified, stabilized version has been proposed [12] with extensions to quantify its Monte Carlo error in phylogenetics [15]. PS is shown to outperform the other methods across all scenarios, remaining well-behaved in cases with high dimensions where all three IS methods fail, even when these IS methods use a huge number of posterior samples [8].

Recently, Xie et al. [14] introduced stepping-stone sampling (SS), which employs ideas from both importance sampling and path sampling to estimate the marginal likelihood using a path that bridges the posterior and prior distribution of a model. Using a Gaussian model example, the authors demonstrate that SS yields a substantially less biased estimator than PS, while requiring significantly fewer path steps (called ratios in the context of SS) to reliably estimate the marginal likelihood. Further, SS outperforms the HME in terms of accuracy, consistency and repeatability [16]. While the performance of PS and SS was originally assessed using Gaussian model examples and small phylogenetic examples, these approaches have recently been shown to considerably outperform the HME and a posterior simulation-based analogue of Akaike’s information criterion (AICM) using extensive simulations and empirical analyses in the context of demographic and relaxed molecular clock model comparison [17].

As a consequence of these developments, path sampling (PS; [8]) and stepping-stone sampling (SS; [14]) estimators are currently being integrated in popular software packages such as BEAST [18]. These methods represent very general estimators as they can be applied to any model for which MCMC samples can be obtained. Despite the increased computational demands associated with these estimators, they are particularly suited to assess the performance of high-dimensional models, since the HME systematically favors parameter-rich models (see the corresponding section in this paper) [8].

In this paper, we focus on direct (log) Bayes factor estimation between two competing models instead of estimating each individual model’s marginal likelihood. Direct (log) Bayes factor estimation, available in the path sampling paradigm as ‘model-switch path sampling’ and connecting the two models under comparison in the space of unnormalized densities, is more accurate and less prone to errors as we show here, which is especially important for cases where the difference between the logarithm of the marginal likelihoods of the two models is small compared to the separate marginal likelihoods themselves [8]. Given the recent introduction of stepping-stone sampling to estimate marginal likelihoods more efficiently [14], we here introduce ‘model-switch stepping-stone sampling’ to perform direct (log) Bayes factor estimation and provide evidence that this represents the more reliable approach.

We test the mentioned approaches on three data sets (see Methods section) with distinctive properties to show the advantages of our presented approach. Two mammalian data sets are analyzed, which are expected to yield increases in model fit due to the CpG-methylation-deamination process acting upon mammalian sequences and the ability of the proposed context-dependent model to incorporate this process. The largest of these data sets contains large amounts of sites for each of the parameters that need to be estimated and is hence expected to yield accurate context-dependent parameter estimates that will lead to a substantial increase in model fit over a traditional site-independent model. The smaller mammalian data set is likely to yield context-dependent parameter estimates with large variance, rendering the process of assessing differences in model fit challenging and hence presenting an interesting case for the different model selection approaches that are being compared. Finally, we test a green plant data set of which the substitution processes are not expected to conform to those accounted for by the context-dependent model and which is hence expected to yield a decrease in model fit compared to a site-independent evolutionary model.

## Methods

### Data

A first (mammalian) data set is a subset of the sequence data set analyzed in Prasad et al. [19], which is an expanded version of that reported by Thomas et al. (2003). All sequences are orthologous to a 1.9-Mb region of human chromosome 7 (build hg18, chr7:115,597,757-117,475,182) that includes 10 known genes (e.g., CFTR, ST7, and CAV1). We selected five sequences from the Laurasiatheria, i.e. the sequences of domestic pig (*Sus scrofa domestica*), Indian muntjac (*Muntiacus muntjak vaginalis*), sheep (*Ovis aries*), cow (*Bos taurus*) and horse (*Equus caballus*) assuming the following topology ((((cow,sheep),muntjak),pig),horse). The reported analyses in this manuscript were performed on the conserved non-coding part of the data set of Prasad et al. [19], comprising 97,682 nucleotides per sequence. We refer to this data set as the “Laurasiatheria” data set.

A second (mammalian) data set consists of the *ψη*-globin pseudogene sequences of six primates: human (*Homo Sapiens*), chimpanzee (*Pan Troglodytes*), gorilla (*Gorilla Gorilla*), orang-utan (*Pongo Pygmaeus*), rhesus monkey (*Macaca Mulatta*) and spider monkey (*Ateles Geoffroyi*), containing 6,166 nucleotides in each sequence. We have used the fixed consensus tree shown in the work of Yang [20] and refer to this dataset as the “Pseudogenes” data set.

A third (plant) data set consists of 20 small subunit (SSU) rRNA genes (nuclear) obtained from the alignment of Karol et al. [21]. We have used the following sequences: *Cyanophora paradoxa*, *Nephroselmis olivacea*, *Chlamydomonas moewusii*, *Volvox carteri*, *Paulschulzia pseudovolvox*, *Coleochaete orbicularis 2651*, *Coleochaete solute 32d1*, *Coleochaete irregularis 3d2*, *Coleochaete sieminskiana 10d1*, *Zygnema peliosporum*, *Mougeotia sp758*, *Gonatozygon monotaenium 1253*, *Onychonema sp832*, *Cosmocladium perissum 2447*, *Lychnothamnus barbatus 159*, *Nitellopsis obtusa F131B*, *Chara connivens F140*, *Lamprothamnium macropogon X695*, *Arabidopsis thaliana* and *Taxus mairei*. We used the 50% majority-rule posterior consensus tree under the general time-reversible model. We refer to this dataset as the “Nuclear SSU rRNA” data set, which contains 1,619 nucleotides per sequence.

### Context-dependent evolutionary model

We allow the substitution probabilities for a given site to depend on its “evolutionary context”, i.e. the combination of neighboring bases of that site. We assume that evolution occurs independently on each tree branch (see e.g. [22]) but do not employ a branch-specific (or lineage-specific) context-dependent evolutionary model. We do not follow the approach of Hwang and Green [23], who have approximated the continuous-time Markov substitution process by partitioning each branch into two or more discrete time units so that the average substitution rate per time unit is ≤ 0.005, because our previous work demonstrated that this does not result in different parameter estimates [24]. We here present a first-order (i.e. depending on the two immediate neighbors) context-dependent evolutionary model which mimics the model of Hwang and Green [23].

For bases *x*≠*z* in the first-order context-dependent model, let *ψ*_*i*_(*x*→*z*∣*wy*) be the probability that, in one unit of time, the base *x* at position *i* mutates to *z*, given neighboring bases *w* (at position *i*−1) and *y* (at position *i*+1). Should the branch not be partitioned, it is hence assumed that the neighboring bases of position *i* remain constant across the branch. Partitioning the branch into two or more parts allows the substitution probabilities to depend on more recent ancestral sequences than at the start of the branch. The probability of no substitution is $\psi_{i}(x\to x|\text{wy})=1-\underset{z\neq x}{\sum }\psi_{i}(x\to z|\text{wy})$. If *w*, *x*, *y* or *z* is a gap, *ψ*_*i*_(*x*→*z*∣*wy*)=1. Each combination of two neighbors yields its own set of context-dependent frequencies *f*_*w*−*y*_, i.e. the substitution probabilities for a given set of immediate neighbors *w* and *y* yield a distribution of frequencies for those neighbors. These frequencies are used to scale the substitution probabilities *ψ*_*i*_(*x*→*z*∣*wy*) so that one unit of time is the time in which we expect to see one change per base, and this for each of the 16 evolutionary contexts, so that $\underset{x}{\sum }\underset{z}{\sum }f_{\text{wxy}}\psi_{i}(x\to z|\text{wy})=1$. This first-order context-dependent substitution model requires 192 parameters to be estimated when it is assumed that complementary events are unequal. Here, we assume that such events are equal, i.e. that *ψ*_*i*_(*x*→*z*∣*wy*)=*ψ*_*i*_(*x*^*c*^→*z*^*c*^∣*y*^*c*^*w*^*c*^), where *x*^*c*^ denotes the complement of *x*, which reduces the number of parameters for this model to 96.

For the Laurasiatheria data set, the distribution of bases *x* that are at the ancestral root sequence is modeled as an inhomogeneous second-order Markov chain with transition parameters *Π*(*x*∣*v*,*w*), where *v* and *w* are the bases that immediately precede *x* (as in [23]). If *x*, *v* or *w* is a gap, *Π*(*x*∣*v*,*w*)=1. For the pseudogenes data set, an inhomogeneous first-order Markov chain with transition parameters *Π*(*x*∣*v*) is assumed (see [25]), where *v* is the base that immediately precedes *x*. If *x* or *v* is a gap, *Π*(*x*∣*v*)=1. For the nuclear SSU data set [21], an zero-order Markov chain *Π*(*x*) is assumed (see Results section). No symmetry conditions are imposed on the *Π* values.

### Prior distributions

Given the importance of using proper prior distributions (see e.g. [26]), we here provide the priors used in this manuscript. Let *T* be the set of branch lengths with *t*_*b*_(*t*_*b*_≥0) one arbitrary branch length and *μ* a hyperparameter in the prior for *t*_*b*_ in *T*. The following prior distributions *q*(.) were chosen for our analysis, with *Γ*(.) the Gamma function:

$$\begin{array}{ll} t_{b}|\mu & ∼\text{Exponential}(\mu ),q(t_{b}|\mu )\\ & =\frac{1}{\mu }e^{-(1/\mu )t_{b}}\text{for each}t_{b}\text{in}T, \end{array}$$

$$\begin{array}{ll} \mu & ∼\text{Inv - gamma}(2.1,1.1),q(\mu )\\ & =\frac{{\left(1.1\right)}^{\left(2.1\right)}}{\Gamma (2.1)}\mu^{-(2.1+1.1)}e^{-1.1/\mu },\;\mu >0. \end{array}$$

Branch lengths are assumed i.i.d. given *μ*. Dirichlet priors (which are uninformative priors) assign densities to groups of parameters that measure proportions (i.e., parameters that must sum to 1). For each set of model frequencies of which the ancestral root sequence is composed, the following prior distribution is assumed:

$$\Pi_{\text{ROOT}}∼\text{Dirichlet}(1,1,1,1),\;q(\Pi_{\text{ROOT}})=\Gamma (4).$$

The above prior is also assumed for the set of base frequencies of the GTR model, which also specify its stationary distribution. For the model parameters of each context (i.e. neighboring base combination) independently, the following prior distribution is assumed (see [27,28]):

ψ∼Dirichlet(1,1,1,1,1,1,1,1,1,1,1,1),q(ψ)=Γ(12).

When the model allows for the presence of multiple contexts of evolution, each context is assumed to have its own prior, independently of other contexts. The use of a hyperparameter for the branch-length priors is to reduce sensitivity of the posterior to the prior [4,29].

### Harmonic mean estimators

Among the available approaches to calculate the marginal likelihood of a model, the harmonic mean estimator (HME) is by far the most used in the field of phylogenetics, only requiring samples from the posterior distribution. Because HME variance may be infinite, a modified stabilized version (sHME) has been proposed [12]. The HME is however often severely biased and results in overestimating the true marginal likelihood [8,14]. Lartillot and Philippe [8] suggest an intuitive reasoning for this by stating that, if the likelihood is unimodal, the marginal likelihood is more or less the product of two factors: the likelihood reached in the high-likelihood region (the mode height) and the relative size of this region (the mode width, which tends to be smaller for higher dimensional models). Estimators such as the HME have difficulties in assessing the mode width, which is estimated by measuring the relative frequency at which points of the sample fall inside and outside the mode, requiring that a sufficient number of points outside the mode be included in the sample. Lartillot and Philippe [8] state that, in practice, the contrast between the low and the high likelihoods is in general so large that even a posterior sample of astronomical size will be virtually confined within the mode. The estimated frequency at which the low-likelihood region is visited is then 0, which means that, in effect, the HME behaves as if the mode was occupying the entire parameter space, and therefore, completely underestimates the dimensional penalty. As a result, the HME overestimates the marginal likelihood, an overestimation that is more pronounced in the case of higher dimensional models, leading to the HME being biased in favor of such models.

### Path sampling

Path sampling is considered a natural generalization of importance sampling and uses many and continuously connected “bridge” densities (called a “path”) to compute (ratios of) normalizing constants [10]. Path sampling is hence an extension of bridge sampling, which generalizes importance sampling through the use of a single “bridge” density. In a comparative study on a variety of methods for computing Bayes factors, from Laplace approximation to bridge sampling, DiCiccio et al. [30] show that bridge sampling typically provides an order of magnitude of improvement. Path sampling, which was not part of their study, has been demonstrated to yield even more dramatic improvement [10].

Using the notation put forward by [8], suppose there are two unnormalized densities, *q*_0_(*θ*) and *q*_1_(*θ*), defined on the same parameter space *Θ*, with corresponding true probability densities

(2) $$p_{i}(\theta )=\frac{1}{Z_{i}}q_{i}(\theta ),i=0,1,$$

where the normalizing constants are

(3) $$Z_{i}=\int_{\Theta }q_{i}(\theta )\mathrm{d\theta},i=0,1.$$

Monte Carlo simulation is widely used in statistics, mainly because of its general applicability, to approximate such analytically intractable normalizing constants. Arguably, it is also the only general method available for dealing with complex, high-dimensional problems [10]. Up until the introduction of bridge sampling and path sampling, estimation methods in statistics often relied on the scheme of importance sampling, either using draws from an approximate density or from one of *p*_*i*_(*θ*). Theoretical [11] and empirical evidence [30,31] provided in the context of bridge sampling, show that substantial reductions of Monte Carlo errors can be achieved with little or minor increase in computational effort, by using draws from more than one *p*_*i*_(*θ*). The key idea is to use “bridge” densities to effectively shorten the distances among target densities, distances that are responsible for large Monte Carlo errors with the standard importance sampling methods. In fact, Gelman and Meng [10] show that importance sampling, bridge sampling and path sampling represent a natural methodological evolution, from using no bridge densities to using a (potentially) infinite number of them.

Lartillot and Philippe [8] recently introduced path sampling in the field of phylogenetics and propose a continuous method to directly estimate log Bayes factors, which has the advantage of yielding greater accuracy compared to a previously introduced discrete method [32]. This approach (called “model-switch” path sampling) considers the unnormalized density function *q*_*β*_ to constitute a direct path between the two competing models [8], which has normalizing constant *c*_*β*_ yielding the normalized density *p*_*β*_. In other words, *β* interpolates between the two models’ posterior densities. The originally proposed continuous method consists in equilibrating a MCMC under *β*=0, followed by smoothly increasing the value of *β*, by adding a constant increment *δβ* after each series of *Q* cycles, until *β*=1 is reached. During this procedure, points *θ*_*k*_ are saved before each update of *β*. Denote (*β*_*k*_,*θ*_*k*_)_*k*=0..*K*_ the series of points obtained this way. The constant-increment approach of Lartillot and Philippe [8] assumes in particular *β*_0_=0, *β*_*K*_=1, and ∀*k*,0≤*k*<*K*,*β*_*k*+1_−*β*_*k*_=*δβ*, which is reflected in the expressions for the continuous estimator and its corresponding discretization and sampling error. Specifically, the estimate of

(4) $$\text{ln}Z_{1}-\text{ln}Z_{0}=\int_{0}^{1}E_{\beta }[U(\theta )]\mathrm{d\beta},$$

with *Z*_*i*_ the normalizing constant of model *i*, is given by:

(5) $${\hat{\mu }}_{\text{qs}}=\frac{1}{K}\left(\frac{1}{2}U(\theta_{0})+\overset{K-1}{\underset{k=1}{\sum }}U(\theta_{k})+\frac{1}{2}U(\theta_{K})\right),$$

with

(6) $$\begin{array}{ll} U(\theta )= & \text{ln}f(y|\theta ,M_{1})+\text{ln}\Pi (\theta |M_{1})-\text{ln}f(y|\theta ,M_{0})\\ & -\text{ln}\Pi (\theta |M_{0}), \end{array}$$

where *y* represents the data (e.g., nucleotide sequences), *Θ* is the vector of model parameters, *M*_*i*_,*i*=0,1 are the two models under consideration, *f*(*y*∣*θ*,*M*) is the likelihood function and *Π*(*θ*∣*M*_*i*_),*i*=0,1 the model priors. As the authors mention, one can also start at *β*=1, equilibrate the MCMC, and then progressively decrease *β*, while sampling along the path down to *β*=0. The mean of both estimates can then be used as a final estimate of the log Bayes factor (called the bidirectional mean). We here adopt the terminology of Lartillot and Philippe [8] and call the move from *β*=0 to *β*=1 the “annealing” integration whereas the move from *β*=1 to *β*=0 is called the “melting” integration. The constant-increment approach [8] may be considered to yield an oversimplified expression for the continuous estimator. It is based on Simpson’s triangulation formula to calculate the contribution to the overall log Bayes factor of one step of the integration, for example from *β*_*k*_ to *β*_*k*+1_ as follows:

(7) $$(\beta_{k+1}-\beta_{k})\left(\frac{1}{2}U(\theta_{k})+\frac{1}{2}U(\theta_{k+1})\right).$$

Given that the increments are constant, it follows that ∀*k*0≤*k*<*K*,*β*_*k*+1_−*β*_*k*_=1/*K* and hence equation (5) is readily obtained. In the case of non-constant increments, equation (5) for the continuous estimator becomes:

(8) $${\hat{\mu }}_{\text{qs}}=\overset{K-1}{\underset{k=0}{\sum }}(\beta_{k+1}-\beta_{k})\left(\frac{1}{2}U(\theta_{k})+\frac{1}{2}U(\theta_{k+1})\right).$$

Equation 8 is restricted to a sample from the last iteration of each *β* to calculate the marginal likelihood, as in the original paper on path sampling [8]. Therefore, this approach only uses a limited amount of the available samples and more information can potentially be retrieved from the MCMC iterations in order to improve the estimation of the marginal likelihood. One possibility lies in using the mean of multiple values for each *β*, collected at fixed intervals, which requires replacing *U*(*θ*_*k*_) and *U*(*θ*_*k*+1_) in equation 8 by $Û(\theta_{k})$ and $Û(\theta_{k+1})$ respectively, i. e. the mean of a collection of samples at *β*_*k*_ and *β*_*k*+1_ respectively. We propose to collect a sample every 10 update cycles, where an update cycle involves an update for every model parameter, branch length and ancestral site. Such a scenario is valid since, for large values of *K*, $p_{\beta_{k-1}}$ is only slightly more dispersed than $p_{\beta_{k}}$ (i.e. in such a case, the subsequent power posteriors will be very similar) and hence serves as an excellent importance distribution [33]. In other words, for large values of *K*, the MCMC chain will smoothly transition between different values of *β*, and hence the likelihood and parameter values in subsequent MCMC chains will not be all that different from one another, which allows for early sampling at each new value of *β*. We refer to Appendix A for a derivation of the discretization and sampling error corresponding to the path sampling estimator discussed here.

### Stepping-stone sampling

Recently, Xie et al. [14] presented a novel approach to estimate marginal likelihoods called ‘stepping-stone sampling’. The authors show that their approach yields an unbiased estimate of the marginal likelihood, as opposed to PS, and that their calculations can be performed more efficiently than PS. Using a simulated Gaussian example data set, which is instructive because of the fact that the true value of the marginal likelihood is available analytically, Xie et al. [14] show that PS and SS perform much better (with SS being the best) than the HME at estimating the marginal likelihood. The authors go on to analyze a 10-taxon green plant data set using DNA sequences of the chloroplast-encoded large subunit of the RuBisCO gene (rbcL) and establish that PS requires a larger number of power posteriors to be explored compared to SS to overcome its additional bias. Using the HME to estimate the marginal likelihood is reported to yield higher values than using both PS and SS.

We here present an extension of their approach to directly calculate (log) Bayes factors, i.e. the stepping-stone version of model-switch path sampling, with the term “model-switch” indicating that a single path directly connects the two models in the space of unnormalized densities. Whereas such a general approach to directly estimate (log) Bayes factors is relatively new in the field of phylogenetics [8], the idea stems from statistics and was first introduced in the work of Meng and Wong [11], who propose a number of approaches to calculate the ratio of two normalizing constants. Using the notation of [14], consider again the unnormalized density function *q*_*β*_, which constitutes a direct path between the two competing models *M*_0_ and *M*_1_[8] and has normalizing constant *c*_*β*_ yielding the normalized density *p*_*β*_:

(9) $$\begin{array}{ll} q_{\beta }(\theta )= & {\left[f(y|\theta ,M_{0})\Pi (\theta |M_{0})\right]}^{1-\beta }\\ & \times {\left[f(y|\theta ,M_{1})\Pi (\theta |M_{1})\right]}^{\beta }, \end{array}$$

(10) $$p_{\beta }(\theta )=q_{\beta }(\theta )/c_{\beta }(\theta ),$$

(11) $$c_{\beta }=\int_{\Theta }q_{\beta }(\theta )\mathrm{d\theta},$$

where again *y* represents the data (e.g., nucleotide sequences), *Θ* is the vector of model parameters, *M*_*i*_,*i*=0,1 are the two models under consideration, *f*(*y*∣*θ*,*M*) is the likelihood function and *Π*(*θ*∣*M*_*i*_),*i*=0,1 the model priors. The goal is to estimate the ratio *c*_1.0_/*c*_0.0_. Similar to the original stepping-stone method, this ratio can be expressed as a product of *K* ratios:

(12) $$r=\frac{c_{1.0}}{c_{0.0}}=\overset{K}{\underset{k=1}{\prod }}\frac{c_{\beta_{k}}}{c_{\beta_{k-1}}},$$

where 0=*β*_0_<…<*β*_*k*−1_<*β*_*k*_<…<*β*_*K*_=1. Each ratio $c_{\beta_{k}}/c_{\beta_{k-1}}$ can be estimated by importance sampling, using $p_{\beta_{k-1}}$ as the importance sampling density. Because $p_{\beta_{k-1}}$ is only slightly different from $p_{\beta_{k}}$, it serves as an excellent importance distribution. One of the *K* ratios, *r*_*k*_, can thus be expressed as follows:

(13) $$\begin{array}{ll} r_{k} & =\frac{c_{\beta_{k}}}{c_{\beta_{k-1}}}\\ & =\frac{\int q_{\beta_{k}}(\theta )\mathrm{d\theta}}{\int q_{\beta_{k-1}}(\theta )\mathrm{d\theta}}\\ & =\frac{\int \left(\frac{q_{\beta_{k}}(\theta )}{p_{\beta_{k-1}}(\theta )}\right)p_{\beta_{k-1}}(\theta )\mathrm{d\theta}}{\int \left(\frac{q_{\beta_{k-1}}(\theta )}{p_{\beta_{k-1}}(\theta )}\right)p_{\beta_{k-1}}(\theta )\mathrm{d\theta}}\\ & =\frac{\int \left(\frac{q_{\beta_{k}}(\theta )}{q_{\beta_{k-1}}(\theta )/c_{\beta_{k-1}}(\theta )}\right)p_{\beta_{k-1}}(\theta )\mathrm{d\theta}}{\int \left(\frac{q_{\beta_{k-1}}(\theta )}{q_{\beta_{k-1}}(\theta )/c_{\beta_{k-1}}(\theta )}\right)p_{\beta_{k-1}}(\theta )\mathrm{d\theta}}\\ & =\int \left(\frac{q_{\beta_{k}}}{q_{\beta_{k-1}}}\right)p_{\beta_{k-1}}(\theta )\mathrm{d\theta}\\ & =\int \frac{{\left[f(y|\theta ,M_{0})\Pi (\theta |M_{0})\right]}^{1-\beta_{k}}{\left[f(y|\theta ,M_{1})\Pi (\theta |M_{1})\right]}^{\beta_{k}}}{{\left[f(y|\theta ,M_{0})\Pi (\theta |M_{0})\right]}^{1-\beta_{k-1}}{\left[f(y|\theta ,M_{1})\Pi (\theta |M_{1})\right]}^{\beta_{k-1}}}p_{\beta_{k-1}}(\theta )\mathrm{d\theta}\\ & =\int \frac{{\left[f(y|\theta ,M_{1})\Pi (\theta |M_{1})\right]}^{\beta_{k}-\beta_{k-1}}}{{\left[f(y|\theta ,M_{0})\Pi (\theta |M_{0})\right]}^{\beta_{k}-\beta_{k-1}}}p_{\beta_{k-1}}(\theta )\mathrm{d\theta}\\ & =E_{p_{\beta_{k-1}}}\left[{\left(\frac{f(y|\theta ,M_{1})\Pi (\theta |M_{1})}{f(y|\theta ,M_{0})\Pi (\theta |M_{0})}\right)}^{\beta_{k}-\beta_{k-1}}\right]. \end{array}$$

An estimator ${\hat{r}}_{k}$ is constructed using samples *θ*_*k*−1,*i*_(*i*=1,2,…,*n*) from $p_{\beta_{k-1}}$:

(14) $${\hat{r}}_{k}=\frac{1}{n}\overset{n}{\underset{i=1}{\sum }}{\left[\frac{f(y|\theta_{k-1,i},M_{1})\Pi (\theta_{k-1,i}|M_{1})}{f(y|\theta_{k-1,i},M_{0})\Pi (\theta_{k-1,i}|M_{0})}\right]}^{\beta_{k}-\beta_{k-1}}.$$

Numerical stability can be improved by factoring out the largest sampled term *η*_*k*_=max_1≤*i*≤*n*_{*f*(*y*∣*θ*_*k*−1,*i*_,*M*_1_)*Π*(*θ*_*k*−1,*i*_∣*M*_1_)/*f*(*y*∣*θ*_*k*−1,*i*_,*M*_0_)*Π*(*θ*_*k*−1,*i*_∣*M*_0_)}:

(15) $$\begin{array}{ll} {\hat{r}}_{k}= & \frac{1}{n}{\left(\eta_{k}\right)}^{\beta_{k}-\beta_{k-1}}\overset{n}{\underset{i=1}{\sum }}\\ & \times {\left[\frac{f(y|\theta_{k-1,i},M_{1})\Pi (\theta_{k-1,i}|M_{1})}{\eta_{k}f(y|\theta_{k-1,i},M_{0})\Pi (\theta_{k-1,i}|M_{0})}\right]}^{\beta_{k}-\beta_{k-1}}. \end{array}$$

Combining all *K* ratios, the SS estimate of the Bayes factor is

(16) $$\hat{r}=\overset{K}{\underset{k=1}{\prod }}{\hat{r}}_{k}.$$

As for the marginal likelihood estimator based on stepping-stone sampling [14], $\hat{r}$ is unbiased, being a product of independent unbiased estimators. On the log scale, and by performing calculations:

(17) $$\begin{array}{rll} \log & {\hat{r}}_{k}\; & \;\\ \;= & (\beta_{k}-\beta_{k-1})\log\eta_{k}\; & \;\\ \; & +\log\left(\frac{1}{n}\overset{n}{\underset{i=1}{\sum }}{\left[\frac{f(y|\theta_{k-1,i},M_{1})\Pi (\theta_{k-1,i}|M_{1})}{\eta_{k}f(y|\theta_{k-1,i},M_{0})\Pi (\theta_{k-1,i}|M_{0})}\right]}^{\beta_{k}-\beta_{k-1}}\right).\; \end{array}$$

Finally, summing $\log{\hat{r}}_{k}$ over all *K* ratios yields the overall estimator:

(18) $$\begin{array}{ll} \log\hat{r} & =\overset{K}{\underset{k=1}{\sum }}\log{\hat{r}}_{k}\\ & =\overset{K}{\underset{k=1}{\sum }}[(\beta_{k}-\beta_{k-1})\log\eta_{k}]\\ & +\overset{K}{\underset{k=1}{\sum }}\log\left(\frac{1}{n}\overset{n}{\underset{i=1}{\sum }}\right)\\ & \left(\times {\left[\frac{f(y|\theta_{k-1,i},M_{1})\Pi (\theta_{k-1,i}|M_{1})}{\eta_{k}f(y|\theta_{k-1,i},M_{0})\Pi (\theta_{k-1,i}|M_{0})}\right]}^{\beta_{k}-\beta_{k-1}}\right). \end{array}$$

Although $\hat{r}$ is unbiased, changing to the log scale introduces a bias [14]. Note that direct Bayes factor estimation (i.e. constructing a path between two competing models) using stepping-stone sampling yields lower variance than calculating the ratio of two independently estimated marginal likelihoods (with each marginal likelihood estimator constructing a path between its prior and posterior). We refer to Appendix B for the derivation of this variance and its comparison to the ratio of variances accompanying marginal likelihood estimation.

### Error assessment

Instead of investigating the discretization and sampling error (the two types of error that occur when performing path sampling; [8]), we here focus on the differences that may occur between annealing and melting versions of the model-switch integrations that yield the log Bayes factor. We use the split-calculation approach introduced in previous work [34], which allows the integration shown in equation 4 to be rewritten as

(19) $$\begin{array}{ll} \int_{0}^{1}E_{\beta }[U(\theta )]\mathrm{d\beta}= & \int_{0}^{\alpha_{1}}E_{\beta }[U(\theta )]\mathrm{d\beta}+\ldots \\ & +\int_{\alpha_{n}}^{1}E_{\beta }[U(\theta )]\mathrm{d\beta}, \end{array}$$

with *α*_0_=0<*α*_1_<…<*α*_*n*_<1=*α*_*n*+1_ dividing the interval [0,1] into *n* subintervals. Each of these integrals can be calculated independently in parallel, allowing the calculation of the log Bayes factor to be distributed across multiple computer nodes (20 in this case), yielding results much faster than when running on a single node. While the method discussed in [34] was applied to path sampling, it can easily be adapted for stepping-stone sampling (not shown) and hence each integral shown in equation 19 can be calculated using both path sampling and stepping-stone sampling. We calculate the difference in contribution to the log Bayes factor for each of the calculated 20 sub-integrals and consider the sum of the absolute values of these differences to be a bidirectional error (or repeatability error). The higher this error, the larger the difference between both annealing and melting calculations of the same sub-integral, indicating that more stringent computational settings are needed to accurately estimate the contribution to the total log Bayes factor.

Even using very demanding computational settings to calculate the log Bayes factor in both directions, small differences between the two estimates are to be expected for two reasons. One is the repeatability issue discussed in [16], which results in small deviations of the marginal likelihood depending on the starting seeds of the analyses, a second is the so-called “thermic lag” of the MCMC chain [8]. Indeed, as *β* changes continuously during sampling, the chain is never exactly at equilibrium, which will cause a “thermic lag” of the MCMC chain. When sampling a value of *Θ* at the current value of *β*, one is in effect sampling from $p_{\beta^{\prime }}$, with *β*^′^ slightly smaller than *β*. Because *U*(*θ*) is an increasing function of *β*, one expects this lag to result on average in an underestimation of the true marginal likelihood when *β* goes from 0 to 1 (i.e. the ‘annealing’ integration) and in an overestimation when *β* goes from 1 to 0 (i.e. the ‘melting’ integration) [8]. This thermic lag bias results from the fact that when the value of *β* is adjusted, the Markov chain takes some time to adjust to the new value [14], i.e. needs to be equilibrated before taking samples. We here check how consistent the estimators are in yielding an underestimation for the annealing calculations and an overestimation for the melting calculations.

## Results

### Laurasiatheria data set

As a means of comparison for our proposed approach, we first estimate the marginal likelihood of the presented context-dependent model and a site-independent reference model known as the general time-reversible (GTR) evolutionary model, which contains 5 free evolutionary parameters and 3 free base frequencies. The HME and sHME estimates of the marginal likelihood for these models are listed in Table 1, showing that (according to the HME and sHME) the context-dependent model (HG04) offers a drastic improvement in model fit over the general time-reversible model, with a log Bayes factor of 3522.93 log units. However, as mentioned in the Methods section, the HME tends to be biased towards higher-dimensional models, meaning that the log Bayes factor shown in Table 1 is possibly an overestimation of the true log Bayes factor.

**Table 1:** Model comparison using the harmonic mean estimator (HME) for each of the three data sets

**Data set**	**Model**	**Root order**	**HME**	**sHME**	**log BF**
Laurasiatheria	GTR	Independence (Second)	-173720.23	-173342.71	-
	HG04	Second	-170093.65	-169819.78	3522.93
Pseudogenes	GTR	Independence (First)	-14026.11	-14001.45	-
	HG04	First	-13772.80	-13738.83	262.62
Nuclear SSU rRNA	GTR	Independence (Zero)	-8441.69	-8409.53	-
	HG04	Zero	-8412.27	-8374.33	35.20
	GTR	Independence (First)	-8435.35	-8409.82	-
	HG04	First	-8395.11	-8364.34	45.48
	GTR	Independence (Second)	-8445.00	-8408.39	-
	HG04	Second	-8399.92	-8353.75	54.64

Harmonic mean estimates (HME) and stabilized harmonic mean estimates (sHME) for both the site-independent general time-reversible model (GTR) and the context-dependent model of Hwang and Green (HG04) [23], combined with the one or more ancestral root distributions [24]. Great care needs to be taken to make sure that under the GTR model, the same sites are taken into account when calculating the likelihood, this due to the presence of gaps. We emphasize this by stating which ancestral root distribution is used for the GTR model, on top of eliminating those sites for which the dependency pattern contains one or more gaps. The proposed context-dependent model (HG04) clearly outperforms the site-independent GTR model for all the data sets, according to the HME and sHME. Further, for the nuclear SSU rRNA data set and according to the sHME, the difference in model fit increases in favor of the HG04 model with increasing dependencies at the ancestral root.

As a baseline for the comparison of our analyses of different sigmoid shape parameters to determine which power posteriors to estimate, we first used a flexible-increment approach as introduced in previous work [34] and determined the annealing and melting estimates for the log Bayes factor and the accompanying bidirectional error (see Methods section). The flexible-increment approach is an extension of the original (constant-increment) path sampling method, where each integration interval shown in equation 19 employs a different but constant increment size for *β*. An equal number of iterations were run across all 20 integration intervals, summing up to a total of *K*=2.000 path steps, with *Q*=200 MCMC iterations being run per path step (see Additional file 1: Table S1, settings ‘F’ - ‘2.000’ - ‘200’). Our flexible-increment approach yields lower bidirectional errors for all three log Bayes factor estimators compared to the original path sampling method [8] (see Additional file 1: Table S1, settings ‘C’ - ‘2.000’ - ‘200’) and yields more similar results for the annealing and melting integrations. Using these settings, it is immediately apparent that the estimated log Bayes factor using PS or SS is drastically different from the one estimated earlier in the manuscript using the HME and sHME, with the log Bayes factor estimates differing by about 600 log units.

**Figure 1:**
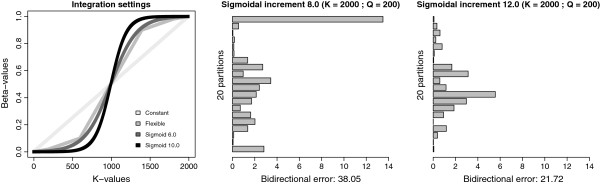
**Sigmoid shape comparison.** Comparison of different integration settings for the three log Bayes factor estimators (left), showing how a sigmoidal shape with *α*=6.0 is closest to our flexible-increment approach, while *α*=10.0 yields a curve that slowly converges towards both ends of the integration interval. The constant-increment approach is clearly a too rude approximation of a path between the two models. Bidirectional errors for a sigmoidal shape of *α*=8.0 (middle), showing that such a curve yields large errors towards both priors and that a higher shape value would be preferred. Bidirectional errors for a sigmoidal shape of *α*=12.0 (right), showing that such a curve yields larger errors in the middle of the integration interval although nowhere near the errors towards the priors for *α*=8.0.

**Figure 2:**
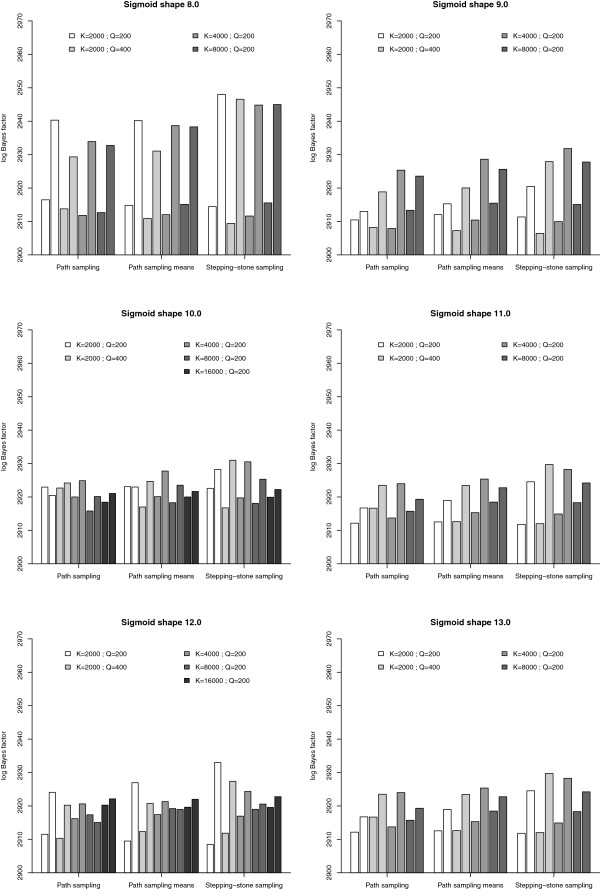
**Model comparison using path sampling (PS) and stepping-stone sampling (SS) for the Laurasiatheria data set.** Laurasiatheria data set: visual comparison of annealing and melting estimates (shown side by side) for the log Bayes factor for different sigmoid shape values. For *α*=10.0 and *α*=12.0, these estimates are available for *K*=2.000(*Q*=200 and *Q*=400),4.000,8.000 and16.000, while for *α*=8.0, *α*=9.0, *α*=11.0 and *α*=13.0 this last value has not been examined. Each subfigure shows annealing and melting estimates for a particular sigmoid shape value, and this for the three estimators discussed: path sampling, the extension of path sampling that uses the mean of a series of samples for each power posterior, and stepping-stone sampling.

**Figure 3:**
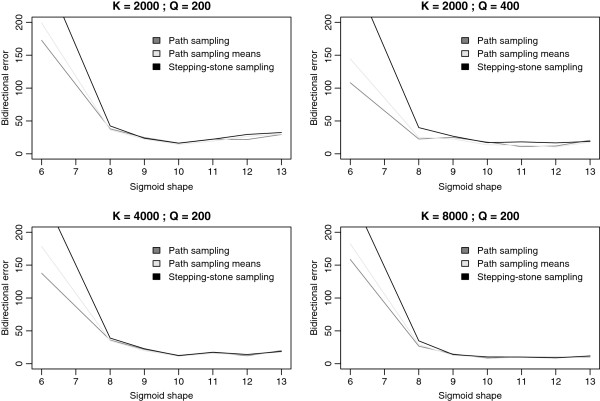
**Comparison of annealing and melting estimates with increasing computational settings.** Laurasiatheria data set: visual comparison of the bidirectional mean log Bayes factor, estimated using stepping-stone sampling, for each sigmoid shape value with the corresponding intervals composed of both annealing and melting estimates. In general, these intervals decrease in width with increasing computational settings.

As discussed earlier, the manual determination and refinement of the number of path steps and MCMC iterations per path step is an iterative and time-consuming process. We used identical integration settings (*K*=2000 and *Q*=200) and estimate the log Bayes factor using a sigmoidal approach to determine the path steps to traverse. Based on a visual comparison (see Figure 1), a shape parameter of *α*=6.0 is an appropriate starting value, which is increased until the accuracy of the results can no longer be improved. Annealing and melting integrations, shown side by side in Figure 2, for the different sigmoid shape values indicate that the most adequate performance is obtained for sigmoid shape values between 10.0 and 12.0, where bracketing the true value of the log Bayes factor becomes more reliable, although there are still quite a few units of difference between them. We increased the shape parameter up to *α*=13.0, revealing that the lowest bidirectional errors are reached for *α*=10.0 (see Figure 3), and this for all three estimators. The computational settings (i.e. for *K* and *Q*) used so far focus on running many short chains, with each subsequent chain only slightly different than the previous one. We now test what the optimal values for both parameters are in order to achieve optimal performance.

Given that Xie et al. [14] have shown that a value of *K*=8 path steps is sufficient for marginal likelihood estimation using stepping-stone sampling for a data set of 10 green plant rbcL sequences (and that *K*=32 is sufficient for path sampling), we have first reversed the integration settings for two shape values (i.e. *K*=200 and *Q*=2.000 for *α*=10.0 and *α*=12.0; see Additional file 1: Table S1). The results seem to indicate that both versions of the path sampling estimator converge really well, with reasonable bidirectional errors. This result is misleading however, as shown by our stepping-stone sampling estimator (which is a less biased estimator) which converges to a different value for the log Bayes factor. In fact, only stepping-stone sampling offers an indication that something is amiss, given its higher bidirectional error, but mainly due to its melting estimate of the log Bayes factor, which is much higher than the annealing estimate. Hence, *K*=200 path steps are clearly insufficient to obtain reliable estimates of the log Bayes factor and may even yield fairly accurate but unreliable results. Increasing *K* to 400 solves the problem of the previous integration settings for a sigmoid shape value *α*=10.0 for all three estimators, albeit that the associated bidirectional error estimates are very large. However, for a shape value *α*=12.0, both path sampling estimators are still unable to bracket the true log Bayes factor value, hence requiring that the number of path steps *K* be further increased. In other words, even a number of path steps of *K*=400, which seems to be excessive based on the work of Xie et al. [14], is only sufficient when a suitable sigmoid shape value is chosen.

Since doubling the number of iterations per path step to *Q*=400, when *K*=2.000, only leads to limited improvements of the bidirectional error (see Figure 2 and Additional file 1: Table S1), we now gradually increase the number of path steps, starting with *K*=4.000 or twice the amount of our first series of analyses, since increasing *K* alleviates the bias of stepping-stone sampling [14]. Further increasing the number of path steps to *K*=8.000 confirms this conclusion. Because of the computational demands, we only apply a final doubling of the number of path steps (*K*=16.000) to the sigmoid shape values *α*=10.0 and *α*=12.0 (see Figure 2). Bidirectional errors continue to decrease, leading to even better bracketing of the true value of the log Bayes factor (see Figure 3). Further, comparing the estimates for both sigmoid shapes shows that the different shapes for the first time lead to very similar results for both annealing and melting calculations, allowing us to accurately infer the true value. For *α*=10.0, the mean bidirectional log Bayes factor equals 2921.09 and for *α*=12.0 this equals 2921.16.

**Figure 4:**
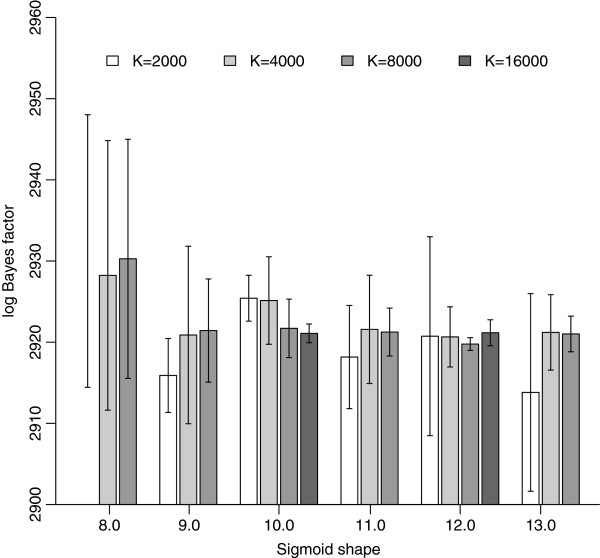
**Bidirectional errors.** Laurasiatheria data set: visual comparison of the bidirectional errors associated with each sigmoid shape value for the three estimators presented in this manuscript. It can be seen that sigmoid shape values between 10.0 and 12.0 are preferred for the Laurasiatheria data set.

Our exploration of different *α* values allows us to create a visual representation for annealing and melting estimates of the log Bayes factor and their bidirectional mean, which is used as the actual estimate of the true value (see Figure 4). For a shape of *α*=12.0, the bidirectional mean is the most stable across the different number of path steps (or ratios) of the different shape values tested and has the appealing property that the difference between annealing and melting estimates decreases the most with increasing path steps (or ratios).

### Pseudogenes data set

We first estimate the marginal likelihood of the HG04 context-dependent model and compared it to that of the GTR model using the HME and sHME (see Table 1). These estimators again report a significant improvement in model fit of the HG04 model, equipped with a first-order ancestral root distribution, over the site-independent GTR model, by a log Bayes factor of 262.62 log units. The much lower number of nucleotides of the pseudogenes data set compared to the Laurasiatheria data set allow for a much quicker evaluation of the (log) Bayes factor using both path sampling approaches as well as the stepping-stone sampling approach. We have therefore chosen to do so using the most stringent settings used for the Laurasiatheria data set, i.e. *K*=16.000 and *Q*=200. The results are shown in Table 2.

**Table 2:** Model comparison using path sampling (PS) and stepping-stone sampling (SS) for the pseudogenes data set

***α***	***K***	***Q***	**BDE(PS)**	**BDE(**$\bar{\text{PS}}$**)**	**BDE(SS)**	**PS-A**	**PS-M**	$\bar{\text{PS}}-\text{A}$	$\bar{\text{PS}}\text{-M}$	**SS-A**	**SS-M**
6.0	16.000	200	8.54	7.74	12.47	174.61	180.14	172.89	179.06	171.26	182.21
8.0			3.80	2.93	3.04	174.75	175.00	174.38	174.72	174.17	175.14
9.0			2.35	2.36	2.45	174.99	174.96	174.82	175.38	174.71	175.52
10.0			2.69	2.65	2.64	174.64	174.98	174.41	175.56	174.35	175.70
11.0			3.49	3.93	3.99	175.66	175.72	175.61	175.89	175.64	176.01
12.0			3.65	2.91	2.86	174.50	174.29	174.57	174.51	174.53	174.64
13.0			2.86	2.59	2.60	175.48	174.79	175.45	174.79	175.40	174.93

Pseudogenes data set. Log Bayes factor estimates for thendent model compared to the site-independent model using regular path sampling (PS; only using the last sample for each path step), path sampling using the mean of a collection of samples from each path step ($\bar{\text{PS}}$) and regular stepping-stone sampling (SS). Bidirectional checks, consisting of annealing (-A) and melting (-M) integrations, were performed for each log Bayes factor calculation, yielding a bidirectional error (BDE) for each estimator. *α* values indicate the shape of the sigmoid function used to construct a path between the two models. *K* indicates the number of path steps (or ratios for SS) used, while *Q* indicates the number of iterations to be performed per path step (or ratio for SS).

Of the different sigmoid shape values tested, a shape value of *α*=6.0 performs much worse than all other values tested, which perform quite similarly. The original path sampling method [8] performed the worst (3 cases) in terms of yielding an underestimation of the log Bayes factor using the annealing integration and an overestimation using the melting integration, with our adaptation of path sampling and our proposed stepping-stone sampling approaching only reporting 1 comparison for which this is the case (i.e. for *α*=13.0). A shape value of *α*=9.0 yields the lowest bidirectional error for SS, yielding a mean bidirectional log Bayes factor of 175.12, confirming the conclusion of the HME and sHME that the proposed context-dependent model HG04 provides a significantly better model fit than the GTR model. Once again however, HME and sHME seem to clearly be biased towards higher-dimensional model as the log Bayes factor is again overestimated. This result for the pseudogenes data set confirms that a context-dependent model offers increased performance over site-independent models in mammalian data sets, even in the case of (relatively) short sequences and a small number of sequences.

### Nuclear SSU rRNA data set

A land plant data set, such as the one analyzed here [21], offers a real challenge for marginal likelihood and (log) Bayes factor estimators using the models compared in this manuscript. The HG04 model is aimed at capturing specific context-dependent processes, such as the CpG-methylation-deamination process in mammalian sequences. Since we are unaware of any such processes being present in land plant sequences, we expect that the context-dependent HG04 model will actually be outperformed by the site-independent GTR model in terms of model fit, on the premise that a model selection approach is used that is able to penalize the model for its excessive amount of parameters that are not accompanied by fitting site patterns.

Given the probably low amount of context-dependent substitution processes that are able to significantly increase model fit, the ancestral root distribution will most likely also not contain any dependencies. We now test this assumption using both the HME, sHME and SS approach, comparing a zero-order, first-order and second-order ancestral root distribution to the GTR model (which uses its base frequencies to determine the independent stationary distribution at the ancestral root). Note that Table 1 shows 3 estimates of the HME and sHME for the GTR model, depending on the actual ancestral root distribution used with the context-dependent model to ensure that the same set of likelihood contributions are used for the different models. According to the HME and sHME (see Table 1), the most parameter-rich ancestral root distribution also explains the data the best, with a second-order distribution being preferred with a log Bayes factor of 54.64 log units over the GTR model (45.48 log units for a first-order and 35.20 log units for a zero-order ancestral root distribution). This result is questionable, since the pseudogene mammalian data set (which is a clear example of a data set that should be analyzed using a context-dependent model and accompanying ancestral root distribution) benefits the most from a first-order ancestral root distribution [26].

We now turn our attention to calculating the log Bayes factor using the proposed SS approach. Assuming *K*=2.000 ratios with *Q*=200 iterations each, a zero-order ancestral root distribution yielded a bidirectional mean log Bayes factor of -58.56 log units versus the GTR model, a first-order distribution a bidirectional mean log Bayes factor of -64.21 log units and a second-order distribution a bidirectional mean log Bayes factor of -94.23 log units. The outcome here is much more biologically plausible than what was obtained using the HME and sHME, with no sign of dependencies being detected, not at the ancestral root nor throughout the remainder of the underlying tree. These results hence support the claim that the HME tends to be biased towards higher-dimensional models, both on the level of the context-dependent model and the ancestral root distribution used, and is hence unable to accurately perform model selection (especially for models with higher dimensions).

This data set is comparable with the pseudogenes data set in terms of computational complexity, meaning that we have again opted to use the most demanding integration settings for the different PS/SS approaches. The results are shown in Table 3. Of the different sigmoid shape values tested, a shape value of *α*=6.0 again performs worse than all other values tested. A shape value of *α*=10.0 yields the lowest bidirectional error for SS, yielding a mean bidirectional log Bayes factor of -59.18, confirming the conclusion of the previous paragraph with more demanding integration settings. Contrary to the two mammalian data sets in this manuscript, the overestimation of the marginal likelihood for high-dimensional models by the HME and sHME leads to a different conclusion for these estimators compared to PS and SS, showing at PS and SS are able to take into account differences in dimension when estimating (log) Bayes factors, unlike the HME and sHME.

**Table 3:** Model comparison using path sampling (PS) and stepping-stone sampling (SS) for the nuclear SSU rRNA data set

***α***	***K***	***Q***	**BDE(PS)**	**BDE(**$\bar{\text{PS}}$**)**	**BDE(SS)**	**PS-A**	**PS-M**	$\bar{\text{PS}}\text{-A}$	$\bar{\text{PS}}\text{-M}$	**SS-A**	**SS-M**
6.0	16.000	200	6.76	4.38	4.32	-58.33	-59.82	-58.82	-57.56	-59.35	-56.82
8.0			2.12	1.99	1.89	-58.92	-59.14	-58.69	-59.11	-58.69	-58.91
9.0			2.08	1.89	1.83	-59.37	-59.48	-58.92	-59.36	-59.00	-59.23
10.0			2.00	1.66	1.64	-60.47	-59.96	-59.94	-59.47	-59.97	-59.38
11.0			2.60	2.74	2.69	-58.89	-59.15	-58.86	-59.23	-58.89	-59.12
12.0			1.86	1.93	2.04	-59.54	-58.80	-59.55	-58.53	-59.57	-58.43
13.0			2.47	2.12	2.20	-58.58	-59.10	-58.44	-59.00	-58.46	-58.87

Nuclear SSU rRNA data set. Log Bayes factor estimates for the context-dependent model compared to the site-independent model using regular path sampling (PS; only using the last sample for each path step), path sampling using the mean of a collection of samples from each path step ($\bar{\text{PS}}$) and regular stepping-stone sampling (SS). Bidirectional checks, consisting of annealing (-A) and melting (-M) integrations, were performed for each log Bayes factor calculation, yielding a bidirectional error (BDE) for each estimator. *α* values indicate the shape of the sigmoid function used to construct a path between the two models. *K* indicates the number of path steps (or ratios for SS) used, while *Q* indicates the number of iterations to be performed per path step (or ratio for SS).

## Discussion and conclusion

In this paper we have compared the performance of two versions of path sampling, an accurate (in that it is able to reliably estimate marginal likelihoods and hence Bayes factors) but computationally demanding model comparison approach, with that of stepping-stone sampling, for which we provide a so-called model-switch version to directly estimate (log) Bayes factors. We have shown that our adaptation of stepping-stone sampling for direct (log) Bayes factor calculation outperforms the original path sampling approach as well as an extension that exploits more samples. Further, we have demonstrated that the (log) Bayes factor estimator proposed in this manuscript generally has lower variance than the (log) Bayes factor estimator obtained through the ratio of marginal likelihoods estimated using stepping-stone sampling.

The large number of combinations of number of path steps / ratios and chain lengths we investigated leads to the recommendation of stepping-stone sampling over path sampling. Indeed, for a relatively small number of path steps / ratios, path sampling tends to converge towards an entirely different value for the (log) Bayes factor, whereas only stepping-stone sampling is able to provide indications that more stringent analyses need to be performed to better approximate the (log) Bayes factor. Stepping-stone sampling is hence better-suited to provide rough initial estimates, using shorter and hence less time-consuming runs, of the magnitude of modeling assumptions. Both path sampling and stepping-stone sampling methods to estimate (log) marginal likelihoods and (log) Bayes factors are much more reliable approaches to perform model selection than the harmonic mean estimator, which is often employed because of its simplicity and computationally appealing properties.

Given that at both ends of the integration interval when performing model-switch path sampling and stepping-stone sampling, one of the models requires sampling from its prior distribution, we have opted for a sigmoid function to determine the necessary power posterior distributions from which sampling is required. Of the shape values compared for the different data sets, a value of between 9.0 and 12.0 is the most appropriate to accurately determine the difference in model fit for high-dimensional models as it is able to accurately bracket the (log) Bayes factor and is accompanied by a low bidirectional error.

Given that path sampling and stepping-stone sampling are far more reliable approaches when estimating the (log) marginal likelihood, we have checked whether this affects the outcome when performing model comparison. Whereas for the Laurasiatheria data set, there is a large difference in the log Bayes factor estimated by path sampling and stepping-stone sampling on one hand, and the harmonic mean estimator on the other hand, these approaches still reach the same conclusion, i.e. that the context-dependent model presented here yields a much better model fit than a site-independent evolutionary model. While this is also the case for a smaller mammalian pseudogene data set, albeit with lower log Bayes factors, we show that for a plant nuclear rRNA SSU data set, the conclusions of the harmonic mean estimator and the path sampling and stepping-stone sampling estimators do not concur, with the former method being unable to accurately penalize the context-dependent model for its excess parameters.

High-dimensional models are typically used in, for example, studies of context-dependence in mammalian sequences. Context-dependent models have been shown to yield much larger increases in model fit than the assumption of among-site rate variation [35] or using mixture models [25]. The so-called first-order context-dependent evolutionary model (i.e. assuming an influence from its two immediate flanking bases), which we analyze in this paper, offers a fair balance between parameter complexity and performance, with further improvements in model fit appearing quite challenging [25]. In other words, the drastic increase in number of parameters, from 12 to 96, is justified by the context-dependent evolutionary processes present in the data, even though this means that far less data per parameter is available. In non-mammalian sequences, these models may be prove to be less useful, as demonstrated in this manuscript, as the drastic increase in number of parameters is not accompanied by fitting context-dependent evolutionary patterns in the underlying data.

While stepping-stone sampling outperforms both versions of path sampling, as we have shown in this paper, it is not a silver bullet and still requires massive computation times, especially for the high-dimensional models tested in this manuscript. Despite recent claims that data augmentation can speed up (log) Bayes factor calculation [36], the use of data augmentation is often a prerequisite to evaluate the likelihood for a context-dependent model and hence does not yield additional increases in speed here. For the Laurasiatheria data set, the most demanding settings we tested (*K*=16.000 and *Q*=200) require about 13 days of calculation running across 40 cores simultaneously using 8-core Intel(R) Xeon(R) CPU X7560 processors running at 2.27GHz. This yields a total computational effort for this data set of about 4.300 days or close to 12 computation years. The other two data sets analyzed in this manuscript require little more than 1 day of calculation running across 40 cores simultaneously on the reported system.

One way to circumvent the added complexity of model-switch path sampling and stepping-stone sampling is to shorten the path from posterior to prior whilst still calculating the marginal likelihood for each model separately. Recently, Fan et al. [16] propose a more general version of stepping-stone sampling that introduces an arbitrary “working” prior distribution parameterized using MCMC samples from the posterior distribution. The authors show that if this reference distribution exactly equals the posterior distribution, the marginal likelihood can be estimated exactly. The authors show that generalized stepping-stone sampling is considerably more efficient and does not require sampling from distributions close to the true prior, currently still an issue with path sampling and stepping-stone sampling for many models. However, at the moment this method is restricted to evaluations on a fixed phylogenetic tree topology. Integrating over plausible tree topologies complicates generalized stepping-stone sampling because of the need to define a reference distribution for topologies that provides a good approximation to the posterior. However, most context-dependent modeling approaches already make the assumption of a fixed underlying tree to make likelihood calculations feasible, making this last requirement less of an issue. The extension of generalized stepping-stone sampling towards constructing a direct path between two competing models is currently the subject of ongoing work.

## Appendix A: discretization and sampling error for path sampling

The corresponding discretization error for model-switch path sampling, originally provided in [8] for the constant-increment approach, associated with using non-constant increments needs to be reformulated. Since *E*_*β*_[*U*] is a monotonous function of *β*, the worst-case upper (resp. lower) error is given by the area between the piecewise continuous function joining the measured values of *E*_*β*_[*U*] and the upper (resp. lower) step function built from them [8]. Both areas are equal to:

(20) $$\sigma_{d}=|\overset{K-1}{\underset{k=0}{\sum }}\frac{1}{2}(\beta_{k+1}-\beta_{k})(E_{\beta_{k+1}}[U]-E_{\beta_{k}}[U])|.$$

Calculating the sampling error in the case of non-constant increments is slightly more complicated:

(21) $$\begin{array}{ll} V[{\hat{\mu }}_{\text{qs}}]=\frac{1}{4} & \left(\overset{K-1}{\underset{k=0}{\sum }}V\left[\left(\beta_{k+1}-\beta_{k})(U(\theta_{k})+U(\theta_{k+1})\right)\right]\right)\\ & +\overset{K-1}{\underset{k=0}{\sum }}\overset{K-1}{\underset{l=0,l\neq k}{\sum }}\text{Cov}\left[\left(\beta_{k+1}-\beta_{k})(U(\theta_{k}\right)\right)\\ & \left(\left(\;\;+U(\theta_{k+1})),(\beta_{l+1}-\beta_{l})(U(\theta_{l})+U(\theta_{l+1}))\right]\right). \end{array}$$

Assuming independence between the successive points of the chain, the first part of the sum in equation (21) can be written as:

(22) $$\overset{K-1}{\underset{k=0}{\sum }}{\left(\beta_{k+1}-\beta_{k}\right)}^{2}(V[U(\theta_{k})]+V[U(\theta_{k+1})]).$$

Using that same assumption, the second part of the sum (i.e., the covariance) in equation (21) only yields a non-zero contribution to the covariance if *l*=*k*+1 or *l*=*k*−1. This yields the following expression for the sampling variance:

(23) $$\begin{array}{ll} \;V[{\hat{\mu }}_{\text{qs}}]=\frac{1}{4} & \left(\overset{K-1}{\underset{k=0}{\sum }}{\left(\beta_{k+1}-\beta_{k}\right)}^{2}(V[U(\theta_{k})]+V[U(\theta_{k+1})])\right)\\ & \left(+2\overset{K-1}{\underset{k=1}{\sum }}(\beta_{k+1}-\beta_{k})(\beta_{k}-\beta_{k-1})V[U(\theta_{k})]\right). \end{array}$$

Again, the presented formulas are valid only if the points are truly independent draws from their respective distributions. If this is not the case, then a factor *τ*=*K*/*K*_*eff*_ (i.e. the decorrelation time) needs to be taken into account in equation 23, to account for the effective sample size [8]. Given that *β* moves between 0 and 1, the decorrelation time might change. While Lartillot and Philippe [8] did not observe large variations in the decorrelation time for different values of *β* for the models they compared, this is not generally so, as shown in [1].

## Appendix B: variance for stepping-stone sampling

As in the original work on (log) marginal likelihood estimation using stepping-stone sampling, we here provide an expression for the simulation variance of ${\hat{r}}_{k}$ in the context of estimating (log) Bayes factor. The simulation variance of ${\hat{r}}_{k}$ is estimated by

(24) $$\begin{array}{l} \hat{\text{Var}}(\hat{r_{k}})\\ =\frac{1}{n^{2}}\overset{n}{\underset{i=1}{\sum }}{\left({\left[\frac{f(y|\theta_{k-1,i},M_{1})\Pi (\theta_{k-1,i}|M_{1})}{f(y|\theta_{k-1,i},M_{0})\Pi (\theta_{k-1,i}|M_{0})}\right]}^{\beta_{k}-\beta_{k-1}}-{\hat{r}}_{k}\right)}^{2} \end{array}$$

Based on the *δ* method [14,37], the variance of $\log\hat{r}=\overset{K}{\underset{k=1}{\sum }}\log({\hat{r}}_{k})$ is approximated by:

(25) $$\begin{array}{l} \hat{\text{Var}}(\log\hat{r})\\ \approx \overset{K}{\underset{k=1}{\sum }}\frac{1}{{\hat{r}}_{k}^{2}}\hat{\text{Var}}(\hat{r_{k}})\\ =\frac{1}{n^{2}}\overset{K}{\underset{k=1}{\sum }}\frac{1}{{\hat{r}}_{k}^{2}}\overset{n}{\underset{i=1}{\sum }}\\ \;\times {\left({\left[\frac{f(y|\theta_{k-1,i},M_{1})\Pi (\theta_{k-1,i}|M_{1})}{f(y|\theta_{k-1,i},M_{0})\Pi (\theta_{k-1,i}|M_{0})}\right]}^{\beta_{k}-\beta_{k-1}}-{\hat{r}}_{k}\right)}^{2}. \end{array}$$

Note that the two equations above are not used in the remainder of this appendix.

Direct (log) Bayes factor estimation is deemed to be less prone to errors than calculating the ratio of independently estimated marginal likelihoods [8]. Using the *δ* method [37] and assuming independence of ${\hat{r}}_{k},k=1,\ldots ,K$

(26) $$\begin{array}{lll} \hat{\text{Var}}\{\log\hat{r}\} & =\overset{K}{\underset{k=1}{\sum }}\hat{\text{Var}}(\log{\hat{r}}_{k})\; & \;\\ & \approx \overset{K}{\underset{k=1}{\sum }}\frac{\hat{\text{Var}}({\hat{r}}_{k})}{{\hat{r}}_{k}^{2}}\; \end{array}$$

and, also using the *δ* method [37]

(27) $$\hat{\text{Var}}\{\log\hat{r}\}\approx \frac{\hat{\text{Var}}\left(\overset{K}{\underset{k=1}{\prod }}{\hat{r}}_{k}\right)}{{\left(\overset{K}{\underset{k=1}{\prod }}{\hat{r}}_{k}\right)}^{2}}.$$

From equations 26 and 27, it follows that

(28) $$\hat{\text{Var}}\left(\overset{K}{\underset{k=1}{\prod }}{\hat{r}}_{k}\right)\approx {\left(\overset{K}{\underset{k=1}{\prod }}{\hat{r}}_{k}\right)}^{2}\overset{K}{\underset{k=1}{\sum }}\frac{\hat{\text{Var}}\left({\hat{r}}_{k}\right)}{{\hat{r}}_{k}^{2}}.$$

We will use equation 28 together with the general approximation

(29) $$\text{Var}\left(\frac{R}{S}\right)\approx \frac{E{\left(R\right)}^{2}}{E{\left(S\right)}^{2}}\left[\frac{\text{Var}(R)}{E{\left(R\right)}^{2}}-\frac{2\text{Cov}(R,S)}{E(R)E(S)}+\frac{\text{Var}(S)}{E{\left(S\right)}^{2}}\right].$$

In particular, assuming that the models *M*_0_ and *M*_1_ have the same priors, it follows from equation 28 that

(30) $$\begin{array}{l} \hat{\text{Var}}(\hat{r})\\ =\;\frac{{\hat{r}}^{2}}{n}\overset{K}{\underset{k=1}{\sum }}\hat{\text{Var}}\left({\left(\frac{f(y|\theta_{k-1},M_{1})}{f(y|\theta_{k-1},M_{0})}\right)}^{\beta_{k}-\beta_{k-1}}\right)/){\left(\frac{c_{\beta_{k}}}{c_{\beta_{k-1}}}\right)}^{2}\\ =\;{\hat{r}}^{2}\overset{K}{\underset{k=1}{\sum }}\frac{\hat{\text{Var}}{\left(f(y|\theta_{k-1},M_{1})\right)}^{\beta_{k}-\beta_{k-1}}}{n{\left(c_{1,\beta_{k}}/c_{1,\beta_{k-1}}\right)}^{2}}\\ \;+\frac{\hat{\text{Var}}{\left(f(y|\theta_{k-1},M_{0})\right)}^{\beta_{k}-\beta_{k-1}}}{n{\left(c_{0,\beta_{k}}/c_{0,\beta_{k-1}}\right)}^{2}}\\ \;-\frac{2\text{Cov}\left(f{\left(y|\theta_{k-1},M_{1}\right)}^{\beta_{k}-\beta_{k-1}},f{\left(y|\theta_{k-1},M_{0}\right)}^{\beta_{k}-\beta_{k-1}}\right)}{n(c_{1,\beta_{k}}/c_{1,\beta_{k-1}})(c_{0,\beta_{k}}/c_{0,\beta_{k-1}})} \end{array}$$

where *θ*_*k*_ is a random draw from $p_{\beta_{k}}$ and

(31) $$c_{j,\beta }=\int f{\left(y|\theta ,M_{j}\right)}^{\beta }\Pi (\theta |M_{j})\mathrm{d\theta .}$$

We compare this expression to the variance of the Bayes factor obtained by independently calculating its marginal likelihoods using stepping-stone sampling [14]. In that case, and using expression 28,

(32) $$\begin{array}{ll} \hat{\text{Var}}(\hat{r}) & =\hat{\text{Var}}\left(\overset{K}{\underset{k=1}{\prod }}\frac{{\hat{r}}_{k,1}}{{\hat{r}}_{k,0}}\right)\\ & =\;{\hat{r}}^{2}\overset{K}{\underset{k=1}{\sum }}\hat{\text{Var}}\left(\frac{{\hat{r}}_{k,1}}{{\hat{r}}_{k,0}}\right)/){\left(\frac{{\hat{r}}_{k,1}}{{\hat{r}}_{k,0}}\right)}^{2} \end{array}$$

where

(33) $${\hat{r}}_{k,j}=\frac{1}{n}\overset{n}{\underset{i=1}{\sum }}f{\left(y|\theta_{k-1,i},M_{j}\right)}^{\beta_{k}-\beta_{k-1}}.$$

From expression 32, it follows that for sufficiently large *n*, using the unbiasedness of ${\hat{r}}_{k}$ and assuming that the separate chains are independent

(34) $$\begin{array}{ll} \hat{\text{Var}}(\hat{r}) & ={\hat{r}}^{2}\overset{K}{\underset{k=1}{\sum }}\hat{\text{Var}}\left(\frac{{\hat{r}}_{k,1}}{{\hat{r}}_{k,0}}\right)/){\left(\frac{c_{1,\beta_{k}}}{c_{1,\beta_{k-1}}}\frac{c_{0,\beta_{k-1}}}{c_{0,\beta_{k}}}\right)}^{2}\\ & ={\hat{r}}^{2}\overset{K}{\underset{k=1}{\sum }}\frac{\hat{\text{Var}}{\left(f(y|\theta_{k-1},M_{1})\right)}^{\beta_{k}-\beta_{k-1}}}{n{\left(c_{1,\beta_{k}}/c_{1,\beta_{k-1}}\right)}^{2}}\\ & \;+\frac{\hat{\text{Var}}{\left(f(y|\theta_{k-1},M_{0})\right)}^{\beta_{k}-\beta_{k-1}}}{n{\left(c_{0,\beta_{k}}/c_{0,\beta_{k-1}}\right)}^{2}}. \end{array}$$

Comparing expressions 30 and 34 shows that the variance of our direct Bayes factor estimation differs from the variance of the ratio of marginal likelihoods only in the last term of expression 30 (i.e. the “covariance term”). This term is not zero because the densities are evaluated in the same parameter draws. Furthermore, we can logically expect this covariance to be generally positive. This formalizes the idea that direct Bayes factor estimation (i.e. constructing a path between two competing models) yields lower variance than calculating the ratio of two independently estimated marginal likelihoods (with each marginal likelihood estimator constructing a path between its prior and posterior), if the two model priors are the same. From expression 30, it follows that the variance of our proposed approach is finite as long as the denominators are not zero, which is to be expected as otherwise the marginal likelihoods themselves would have to be zero.

## Competing interests

The authors declare that they have no competing interests.

## Authors’ contributions

GB initiated the study, designed the context-dependent evolutionary model and the model-switch stepping-stone sampling approach, implemented the path sampling and stepping-stone sampling approaches, performed all the analyses and wrote a first complete version of the manuscript. PL edited the manuscript. SV contributed statistical expertise to the analyses and edited the manuscript. All authors read and approved the final manuscript.

## Supplementary Material

Additional file 1 Model comparison using path sampling (PS) and stepping-stone sampling (SS) for the Laurasiatheria data set. Click here for file
