# Supplementary material for: Make the most of your samples: Bayes factor estimators for high-dimensional models of sequence evolution
Source: BMC Bioinformatics. 2013 Mar 6;14:85. doi: 10.1186/1471-2105-14-85 (PMC3651733; doi:10.1186/1471-2105-14-85)
Supplement: Additional file 1 — Model comparison using path sampling (PS) and stepping-stone sampling (SS) for the Laurasiatheria data set. [file 1471-2105-14-85-S1.pdf]

Additional file

Table S1 - Model comparison using path sampling (PS) and stepping-stone sampling (SS) for the Laurasiatheria data set

Table S1: Laurasiatheria data set. Log Bayes factor estimates for the context-dependent model compared to the site-independent model using regular path sampling (PS; only using the last sample for each path step), path sampling using the mean of a collection of samples from each path step ($\overline{\text{PS}}$) and regular stepping-stone sampling (SS). Bidirectional checks, consisting of annealing (-A) and melting (-M) integrations, were performed for each log Bayes factor calculation, yielding a bidirectional error (BDE) for each estimator. α values indicate the shape of the sigmoid function used to construct a path between the two models, 'C' indicates a constant increment and 'F' indicates the flexible-increment approach (using a constant increment in each sub-integral, but possibly different increments across sub-integrals). K indicates the number of path steps (or ratios for SS) used, while Q indicates the number of iterations to be performed per path step (or ratio for SS). Increasing K is shown to reduce the bidirectional error the most, across different sigmoid shapes, which in turn leads to better converging annealing and melting estimates for the log Bayes factors. Stepping-stone sampling leads to more reliable estimates of the log Bayes factor using a bidirectional check, i.e. the annealing integration yields an underestimation and the melting integration an overestimation of the log Bayes factor on a more consistent basis than both versions of path sampling (a * indicates where this does not hold).

α	K	Q	BDE(PS)	BDE($\overline{\text{PS}}$)	BDE(SS)	PS-A	PS-M	$\overline{\text{PS}}$-A	$\overline{\text{PS}}$-M	SS-A	SS-M
10.0	200	2.000	28.64	11.43	59.99	2872.14*	2868.65*	2862.97	2863.43	2866.44	2926.32
12.0			40.39	24.32	91.64	2844.74	2856.53	2841.04	2860.62	2842.29	2932.64
10.0	400	2.000	514.83	517.90	524.26	2892.36	2979.82	2887.95	2983.15	2888.13	3009.92
12.0			17.60	11.62	35.79	2884.79	2887.00	2886.17*	2883.96*	2886.71	2920.93
C	2.000	200	107.91	132.28	177.37	2891.46	2980.96	2884.84	3001.88	2876.08	3039.59
F			28.81	43.48	42.07	2921.38	2938.53	2920.07	2934.37	2917.94	2953.70
6.0			172.59	199.03	286.21	2861.14	3019.82	2854.86	3041.62	2839.20	3115.79
8.0			38.05	36.43	42.48	2916.48	2940.31	2914.83	2940.18	2914.44	2948.02
9.0			24.80	22.28	23.70	2910.47	2912.99	2912.04	2915.27	2911.35	2920.46
10.0			16.38	14.35	16.18	2922.96*	2920.47*	2923.07*	2922.99*	2922.59	2928.24
11.0			22.43	19.41	22.25	2912.16	2916.71	2912.54	2918.95	2911.81	2924.53
12.0			21.72	26.50	29.48	2911.51	2924.05	2909.50	2926.97	2908.48	2932.99
13.0			29.41	29.91	32.40	2904.38	2914.38	2902.78	2919.17	2901.64	2925.98
6.0	2.000	400	108.09	144.51	282.38	2888.75	2982.60	2874.60	3013.11	2850.22	3128.29
8.0			22.52	25.05	39.91	2913.77	2929.32	2910.88	2931.05	2909.40	2946.58
9.0			24.99	22.55	26.79	2908.19	2918.85	2907.27	2920.04	2906.44	2927.91
10.0			17.94	13.72	17.01	2922.69	2924.19	2917.01	2924.67	2916.75	2930.99
11.0			10.94	13.14	18.18	2916.66	2923.49	2912.62	2923.43	2912.00	2929.71
12.0			11.93	10.46	16.65	2910.31	2920.22	2912.31	2920.75	2911.85	2927.36
13.0			20.63	17.70	18.92	2919.42*	2914.90*	2914.68	2917.90	2914.04	2925.02
6.0	4.000	200	137.70	178.48	261.22	2876.49	3010.66	2864.81	3040.24	2847.86	3107.33
8.0			36.08	35.16	39.06	2911.81	2933.92	2912.04	2938.68	2911.63	2944.83
9.0			21.80	19.73	22.82	2907.90	2925.35	2910.43	2928.63	2909.95	2931.82
10.0			12.77	11.53	12.34	2920.00	2924.88	2920.09	2927.76	2919.74	2930.52
11.0			17.16	15.63	17.51	2913.72	2923.98	2915.30	2925.35	2914.91	2928.25
12.0			12.10	13.94	14.09	2916.20	2920.61	2917.41	2921.28	2916.94	2924.36
13.0			20.08	17.81	18.73	2916.00	2919.52	2917.06	2922.39	2916.56	2925.85
6.0	8.000	200	158.64	182.41	259.74	2872.11	3026.29	2865.01	3042.61	2851.77	3107.52
8.0			26.75	29.09	34.83	2912.66	2932.74	2915.13	2938.31	2915.55	2945.00
9.0			14.77	12.79	14.07	2913.35	2923.57	2915.48	2925.62	2915.08	2927.79
10.0			8.64	9.96	10.39	2915.81	2920.14	2918.28	2923.52	2918.10	2925.31
11.0			10.22	9.71	10.21	2915.71	2919.31	2918.47	2922.75	2918.30	2924.20
12.0			9.93	9.22	8.99	2917.36*	2915.06*	2919.21*	2918.94*	2918.99	2920.55
13.0			10.31	11.22	11.89	2917.72	2920.61	2919.04	2921.48	2918.82	2923.21
10.0	16.000	200	10.49	8.61	8.78	2918.45	2921.06	2920.01	2921.66	2919.93	2922.24
12.0			6.50	6.99	7.55	2920.24	2922.11	2919.63	2921.98	2919.56	2922.76
